# Attributing non-specific symptoms to cancer in general practice: A scoping review

**DOI:** 10.1371/journal.pone.0322264

**Published:** 2025-06-23

**Authors:** Gladys Langue, India Pinker, Valerie Moran, Sophie Pilleron

**Affiliations:** 1 Ageing, Cancer and Disparities Research Unit, Department of Precision Health, Luxembourg Institute of Health, Strassen, Luxembourg; 2 Faculty of Science, Technology and Medicine, University of Luxembourg, Esch-sur-Alzette, Luxembourg; 3 Socio-Economic, Environmental Health & Health Services Research Group, Department of Precision Health, Luxembourg Institute of Health, Strassen, Luxembourg; 4 Socio-Economic, Environmental Health & Health Services Research Group, Living Conditions Department, Luxembourg Institute of Socio-Economic Research, Esch-sur-Alzette, Luxembourg; Virginia Mason Franciscan Health, UNITED STATES OF AMERICA

## Abstract

**Background:**

Non-specific cancer symptoms are challenging to interpret in general practice. They can be attributed to a wide range of other conditions and delay the cancer diagnosis, increasing the risk of poor outcomes.

**Aim:**

To summarise existing knowledge on the attribution of non-specific symptoms to potential cancer in general practice and identify gaps in the literature.

**Method:**

We conducted a scoping review, following Joanna Briggs Institute’s guidance and reported according to the PRISMA for scoping reviews checklist. Non-specific symptoms were defined based on NICE guidelines for suspected cancer. We systematically searched six databases and search engines for original papers, systematic reviews and doctoral theses. Two reviewers independently screened titles, abstracts, full-texts and reference lists. Included articles were then uploaded to the AI-based tool ResearchRabbit to identify further papers. Findings were synthesised using the Refined Andersen Model of Total Patient Delay.

**Results:**

Eight studies were included. These addressed fatigue, recurrent infection, pallor, weight loss, and deep vein thrombosis, with general practitioners (GPs) defining the latter two as cancer-specific. Factors influencing attribution to cancer included pre-existing conditions, the number, type and combination of symptoms as well as GPs’ gut feeling and knowledge, patients’ concern, and the frequency of medical visits.

**Conclusion:**

This scoping review identified a limited number of studies on the attribution of non-specific symptoms to potential cancer in general practice, with symptoms such as pruritus and new-onset diabetes not addressed. It highlights the need for further research into GPs diagnostic reasoning related to non-specific cancer symptoms, especially on under-researched symptoms.

## Introduction

General practitioners (GPs) are often the entry point into the healthcare system. They play a critical role in evaluating signs and symptoms and referring patients for further investigations when a serious disease, such as cancer, is suspected [1].

Despite significant advances in diagnosis and treatment, cancer is amongst the most frequently missed diagnoses [2–4], and remains a global health challenge, with over 20 million new cases and approximately 10 million deaths estimated in 2022 [5]. Early cancer detection is essential but often complicated by the wide range of symptoms cancer can present. These range from specific manifestations, such as postmenopausal vaginal bleeding or new-onset seizures in individuals with no history of epilepsy, to non-specific symptoms (NSS), including fatigue, unintentional weight loss, and appetite loss among others [6,7].

Identifying cancer in the context of NSS is particularly challenging for GPs, even though these symptoms are present in 22% of cancer cases [8]. NSS are frequently associated with a variety of non-cancerous conditions, especially in patients with other conditions like heart failure, chronic obstructive pulmonary disease, or depression, which share symptoms such as loss of appetite and fatigability [3,9–12]. Moreover, some NSS are common in general practice, with fatigue reported by one in four patients [13]. This overlap can delay cancer diagnosis [3,14], resulting in more advanced stages at diagnosis, poorer clinical outcomes and reduced quality of life [12,15–17].

Interestingly, many cancer diagnoses are preceded by an increase in the number of general practice consultations in the months prior, suggesting missed opportunities for earlier detection at the GP level [18–20]. However, the process by which GPs attribute NSS to potential cancer remains underexplored [21,22]. This scoping review aims to provide an overview and summarise the existing scientific literature on the attribution of NSS to cancer in general practice, identify influencing factors, and highlight gaps in the current literature.

## Method

The protocol is available on Open Science Framework (OSF) [23]. This scoping review was prepared using the Preferred Reporting Items for Systematic reviews and Meta-Analyses for scoping reviews (PRISMA-ScR) checklist (S1 Text) and Joanna Briggs Institute’s guideline [24].

Since this scoping review relies exclusively on published articles and grey literature, ethical approval was not required.

### Classification of non-specific symptoms

There is no consensus on a classification of NSS in cancer. To standardise our approach, we have adopted the classification of the National Institute for Health and Care Excellence (NICE) [25]. According to the classification, NSS associated with cancer include fatigue, unexplained weight loss, appetite loss, early satiety, deep vein thrombosis, pallor, new-onset diabetes, night sweats, unexplained fever, persistent or recurrent unexplained infections, and pruritus.

### Literature review

In March 2024, we conducted a comprehensive search for peer-reviewed articles in Medline, Cumulative Index to Nursing and Allied Health Literature (CINAHL), PsycInfo and Embase databases. The search strategy including terms referring to “cancer”, “general practitioner” and “symptom attribution” was reviewed by two librarians (S1–S4 Tables). Following the librarians’ recommendations, we excluded NSS from the search strategy, as pilot testing indicated that these symptoms frequently appear only in the full-text rather than in the title or abstract.

We included original qualitative and quantitative research articles and systematic literature reviews. There was no restriction on the period, geography and language. In July 2024, we also searched Google Scholar and Open Access Theses and Dissertations for doctoral theses published since 2022 (S2 Text and S5 Table). This time range was chosen to capture recent scientific work that had not yet been published.

**Fig 1:**
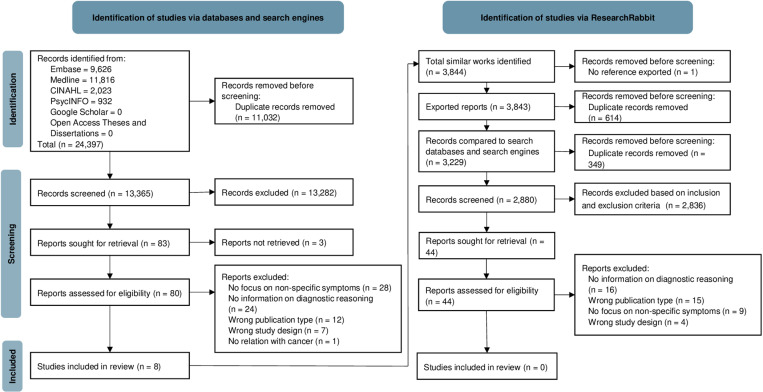
PRISMA flow diagram.

After the removal of duplicates, two independent reviewers screened titles, abstracts, full-texts and the reference lists of the included papers based on predefined eligibility criteria (Table 1). Any disagreements were resolved by a third reviewer. Finally, included articles in the scoping review were uploaded to ResearchRabbit, a publication discovery tool that uses artificial intelligence to identify additional references [26]. This approach aimed to enhance the comprehensiveness of the search*.* Fig 1 shows the selection process.

**Table 1:** Eligibility criteria for the selection of source of evidence.

Component	Inclusion criteria	Exclusion criteria
Population	General practitioners (GP)	Specialist medical doctors Other health professionals (e.g., nurses)
Concept	Non-specific symptoms as defined by NICE Symptom attribution by GPs Suspicion of cancer Justification of cancer suspicion for referral	Symptoms not categorised as non-specific by NICE Symptom attribution by patients Referral for reason other than suspected cancer
Context	Consultation at a primary care level	GP consultation within an emergency ward or other level of care
Study design	Any quantitative, qualitative or mixed-methods design Systematic reviews	Case report Narrative review
Publication type	Peer-reviewed articles Doctoral theses from 2022 onward. The rational for including theses from this period is to capture scientific research results that have not yet been published.	Conference abstracts Editorials Commentaries Letters to editor
Language	No restriction (the authors’ institution hosts diverse language expertise)	None

For each included document, we extracted first author name, year of publication, study objective(s), country(ies) where the study was conducted, type of research, study design, data collection tool, number of GPs and patients and their main characteristics (e.g., sex, years of experience), type of cancer, NSS, factor(s) influencing the symptom attribution and presentation of the factor(s). Data extraction was conducted independently by two reviewers, with discrepancies resolved through discussion between the two reviewers and, if necessary, discussion with a third reviewer. This deviates from the original protocol [23] but was implemented to optimise time and minimise the workload for the reviewers.

Results are summarised using a narrative approach. Factors influencing symptom attribution were categorised according to the Refined Andersen Model of Total Patient Delay [27]. This conceptual framework outlines the various stages a patient may undergo prior to receiving a diagnosis, one of which involves the healthcare professional’s assessment of symptoms. It also identifies the categories of factors that influence this stage, namely disease- (e.g., tumour site), patient- (e.g., demographic), healthcare provider- (e.g., previous experiences) and healthcare system (e.g., healthcare policy) -related factors.

We used EndNote 21.1 to manage references [28] and Rayyan for screening [29].

## Results

Out of 24,397 references, we included eight papers. The list of excluded full-texts with reasons for exclusion is available in S6 and S7 Tables.

No additional articles were found using ResearchRabbit. However, for unknown reasons, it was possible to export only 3,843 of the 3,844 identified references and we could not identify the missing reference.

### Characteristics of included studies (Table 2)

Of the eight included articles [30–37], there were four qualitative studies [30–33], two quantitative studies [34,35], one mixed-method study [36], and one systematic review [37]. Most studies were conducted in Europe [30–35,37], with one study originating from the United States [36]. GPs’ years of practice in the included samples ranged from two to 44 years [30–33,35,36].

**Table 2:** Characteristics of included studies.

First author, Year (reference)	Objective	Country	Method	Symptoms	Cancer type	Category of factor	Factor
**Qualitative design**							
Wagland, 2017 [30]	“explore the views of GPs regarding how best general practice might facilitate timely diagnosis of lung cancer”	United Kingdom	Cross-sectional, focus group discussions with healthcare professionals	Fatigue, recurrent infection, weight loss	Lung cancer	−Disease −Patient −Healthcare provider	−Type of symptom −Consultation frequency −GP’s gut feeling
Saab, 2022 [31]	“This study explored primary healthcare professionals’ experience of referring individuals with signs and symptoms indicative of LC (lung cancer) along the appropriate healthcare pathway and strategies to help primary healthcare professionals detect LC early”	Ireland	Cross-sectional, focus group discussions and interviews	Fatigue, weight loss	Lung cancer	Patient	Pre-existing condition
Barbarit, 2023 [32]	“study how general practitioners conceive the search for cancer in patients with idiopathic venous thromboembolic disease in primary care”	France	Cross-sectional, semi-structured interviews	Deep vein thrombosis	Not specified	Healthcare provider	GP’s medical knowledge
Hajdarevic, 2023 [33]	“This study explored European PCPs’ (primary care physicians) experiences and views on particular cases where they considered that they had been slow to think of, or act on, a possible cancer diagnosis.”	23 European countries	Cross-sectional, survey	Fatigue	Not specified	Patient	Patient’s idea about a benign diagnosis
**Quantitative design**							
Scheel, 2013 [34]	“This article describes: how GPs assess warning signs of cancer and other factors as possible signs of cancer; what actions were taken by GPs; and to what extent a cancer suspicion proved to be correct.”	Norway	Prospective, questionnaire	Fatigue, weight loss	Not specified	Disease	The number, type, and combination of symptoms
Donker, 2016 [35]	“The following questions were investigated 1. What triggers the cancer-related gut feeling of a GP? 2. Based on the gut feeling, which diagnostic actions are taken by the GP? 3. What is the predictive value of cancer-related gut feelings of GPs measured 3 months later? 4. How is the predictive value of GP’s gut feeling influenced by specific triggers, patient and GP characteristics?”	The Netherlands	Prospective, questionnaire	Weight loss	Not specified	Healthcare provider	GP’s gut feeling
**Mixed method**							
Singh, 2012 [36]	“to evaluate whether the SA (situational awareness) framework can be used to analyze outpatient diagnostic errors using a `distributed cognition´ approach that included provider-work system interaction, including interaction with the HER (electronic health records)”	United States	Cross-sectional, medical records review and interviews	Weight loss	Colorectal and lung cancers	Patient	Patient actively reporting a symptom
**Systematic review**							
Friedemann Smith, 2020 [37]	“examine the current evidence regarding GPs’ gut feelings for cancer; collate the factors that are thought to prompt the experience and use of gut feelings, explore how gut feelings are used in primary care; and establish the diagnostic utility of gut feelings through meta-analysis”	United Kingdom, The Netherlands, Denmark, Norway, Sweden, Belgium, Spain	Systematic review and meta-analysis	Pallor, weight loss	Various cancer types	Healthcare provider	−GPs’ knowledge of the patient −Gut feeling

None of included papers explicitly focused on the attribution of NSS to cancer in general practice. While the study by Barbarit and colleagues specifically focused on deep vein thrombosis [32], the remaining seven studies mentioned NSS without making them the primary focus of their investigation.

Weight loss was the most frequently cited NSS [30,31,34–37], followed by fatigue [30,31,33,34]. Recurrent infection and pallor were mentioned in two studies [30,37]. We did not identify any studies mentioning or discussing factors influencing the attribution of new-onset diabetes, pruritus, night sweats, appetite loss, early satiety or unexplained fever to a potential cancer.

A limited number of factors influencing GPs’ symptom attribution were identified, encompassing disease-, patient- and healthcare provider-related factors.

### Disease factors

Not all NSS are regarded equally in the context of cancer suspicion. Despite being classified as NSS, three studies reported that weight loss [30,34] and deep venous thrombosis – particularly when of unclear origin – [32] quickly raised concerns about a potential cancer, even when they occurred in isolation. In contrast, fatigue generally did not elicit concern from GPs unless it was accompanied by other symptoms [30]. In the study conducted by Scheel and colleagues, cancer suspicions were higher with an increasing number of symptoms: GPs suspected cancer in 68 out of 662 (10.3%) fatigue registrations in medical records when fatigue was the sole symptom, whereas the registration of fatigue in combination with at least two warning signs of cancer elicited a suspicion of cancer in 76 out of 137 (55.5%) cases [34].

However, the specific combination of symptoms was also important. Bleeding and fatigue showed a positive association with cancer suspicion (OR = 6.5, 95% Confidence Interval (CI) 1.7 to 24.7) but the combination of fatigue and pain (OR = 0.4, 95% CI 0.2 to 0.7) or that of digestive problem, fatigue, and pain (OR = 0.3, 95% CI 0.0 to 0.8) showed a negative association [34].

### Patient factors

Three studies identified patient-related factors that prompt GPs to suspect cancer [30,31,36] and one highlighted a factor that can deflect suspicion [33].

The way patients communicate their concerns can elicit a suspicion of cancer. In the study of Singh and colleagues, one GP noted that despite documenting the patient’s weight loss in the medical record, he did not recognise its significance until the patient explicitly expressed concern [36].

Conversely, when a patient attributes their symptoms to a benign condition, it may discourage the GP from pursuing further investigation. This was illustrated in the study of Hajdarevic and colleagues, where a GP recounted:

An employee around 60 years at my former clinic came to me with a fatigue. She had been feeling tired from [for] half a year. She had been able to change her work content avoiding activities she felt increased the fatigue. […] Both I as a doctor and she as a patient zoomed in on “burn-out”. [33, p7]

The study of Wagland and colleagues revealed that the frequency of patient consultations may also influence the suspicion of cancer [30]. The authors reported that a patient who rarely visits a GP tends to prompt greater concern from the GP, regardless of the reported symptoms, compared to a patient who consults regularly.

Pre-existing conditions may also contribute to delayed cancer diagnoses, as highlighted by Saab and colleagues [31]. In this study, one GP described attributing a patient’s weight loss to chronic obstructive pulmonary disease rather than considering it as a potential symptom of cancer.

### Healthcare provider factors

The attribution of NSS can also be influenced by medical training, as suggested by the study conducted by Barbarit and colleagues [32]. In this study, GPs acknowledged that their diagnostic reasoning was shaped by their training. Specifically, they reported that an atypical presentation of deep venous thrombosis would automatically raise suspicion of cancer, as this association had been emphasised during their medical education.

GPs familiarity with the patient was also important. The study by Friedemann Smith and colleagues showed that GPs who were well-acquainted with their patients were better positioned to detect changes in appearance, such as alterations in skin tone or weight loss [37].

Three studies examined GPs’ gut feeling [30,35,37]. A gut feeling, when raising concern, was defined as “an uneasy feeling perceived by a GP as he/she is concerned about a possible adverse outcome, even though specific indications are lacking” [38]. It arose particularly in patients presenting with weight loss and a change in their appearance. Although various types of symptoms can trigger gut feelings, interviewed GPs perceive it as particularly valuable when assessing NSS [30].

## Discussion

To our knowledge, this is the first scoping review to explore the attribution of NSS to a potential cancer by GPs. Despite the recognised challenge of NSS in general practice, our scoping review retrieved only eight studies, which suggests that this topic has received limited research attention.

The inherent difficulty in studying diagnostic reasoning may explain the limited number of studies included in our scoping review. Symptom attribution is a critical component of this reasoning and occurs within a GP’s mind. Although some GPs record aspects of their diagnostic reasoning in patient health records, this practice is not standardised and varies widely across practitioners [39,40]. Additionally, GPs may not be aware of all factors influencing their diagnostic reasoning [21]. This makes the systematic study of this cognitive process particularly challenging.

We identified a small number of factors that may influence the suspicion of cancer when a patient presents with NSS. Some factors identified as raising cancer suspicions included specific symptom types or combinations [32,34], patients who rarely consult a GP [30] and the GPs’ gut feeling [30,35,37]. A previous systematic review on cancer-related gut feeling further supports the use of gut feeling as a legitimate criterion for suspected cancer referral [41].

In contrast, pre-existing conditions [31] or the attribution of symptoms to another illness by the patients [33] may lead to cancer not being considered. This highlights the extent to which NSS of cancer can easily be attributed to other conditions. Although factors such as patient sex, history of cancer, symptom duration, GP’s age and patients risk-taking attitude have been identified in the literature as influencing symptom attribution to cancer [34,42], these were not identified in our scoping review. It may be due to our exclusive focus on NSS.

Some NSS listed by NICE are uncommon features of cancer [6,32]. For example, night sweats have a maximum reported prevalence of 6% in patients with Hodgkin lymphoma, while new-onset diabetes is seen only in 1% of pancreatic cancer cases [6]. Due to their low prevalence, these symptoms may be given less priority in research and therefore, be under-recognised by GPs as a potential cancer symptom.

Of the eleven NSS listed by NICE [25], data were available for only five, namely weight loss, fatigue, recurrent infection, pallor and deep venous thrombosis. Among these, weight loss is consistently identified as an alarm symptom, rapidly prompting GPs to consider cancer. This suggests that it may be inappropriate to include weight loss in the list of NSS. This was corroborated during formal discussions with GPs as part of the Public and Patient Involvement activities conducted within the framework of preparing the “Reducing Disparities in Cancer Outcomes – Qualitative component” research project [43]. A deep venous thrombosis, particularly when occurring in atypical contexts, also quickly elicits the suspicion of cancer.

Although included studies have been conducted in different countries, none have provided information on the potential association between the type of healthcare system and the association between NSS and a potential cancer. Consequently, we do not know whether a healthcare system that implements a cancer diagnostic pathway for NSS, such as those in Denmark or United Kingdom [22], facilitates earlier consideration of cancer by GPs compared to a system without these pathways. The existence of these pathways may signify a broader recognition of NSS as being potentially indicative of cancer, thereby encouraging their consideration in clinical practice.

Moreover, despite evidence that comorbidity can delay cancer diagnosis [12,44–46], few studies have investigated GPs’ diagnostic reasoning processes in the context of comorbidity and NSS. Our scoping review identified only one study [31] addressing this issue, yet it offers limited insights, noting only that pre-existing conditions tend to reduce the likelihood of GPs suspecting cancer [31]. However, given population ageing and the rise in the incidence of chronic diseases and cancer [47,48], GPs will increasingly treat multimorbid patients who present with NSS. Advances in cancer treatment have expanded considerably and it is therefore essential to detect new cancer cases as early as possible, so that patients can benefit fully from these advances. Early recognition of NSS as potential signs of cancer is a crucial step in this process. Therefore, understanding how GPs consider the possibility of cancer in these complex cases is essential to support medical doctors in the early detection of cancer.

## Strengths and limitations

The strengths of this scoping review include the absence of restrictions on language and time period, and the search for grey literature. Additionally, we maximised our chances to retrieve relevant papers by utilising ResearchRabbit. Limitations include the lack of standardisation and consistent definition of the concept of “symptom attribution”, which may have reduced the specificity of the chosen keywords. Different classifications of NSS exist in the wider scientific literature, some of which including symptoms beyond those listed by NICE, such as abdominal or back pain, bruising, nausea or vomiting [6,8,49,50]. While NICE’s classification benefits from being developed through expert consensus and provides a clear, structured framework, the origins of other classifications remain unclear. Consequently, papers using NSS symptoms not included in the NICE classification were excluded, potentially excluding relevant literature.

## Conclusion

This scoping review reveals that current knowledge remains limited on the factors influencing diagnostic reasoning in the context of NSS and cancer suspicion in general practice. However, given the increasing prevalence of chronic diseases, this issue is likely to become even more relevant in the coming years, calling for further research on the topic.

Gaining a deeper understanding of how general practitioners interpret and respond to NSS is vital in ensuring that patients are identified and referred appropriately. This need is further underscored by global projections anticipating substantial increases in new cancer cases in the coming years, largely driven by population growth.

Future research should prioritise the investigation of underexplored NSS – such as new-onset diabetes, pruritus, night sweats, loss of appetite, early satiety and unexplained fever – as well as the identification of the factors that most strongly influence diagnostic reasoning. Doing so will help target interventions and research efforts towards where they are most likely to improve clinical practice and support general practitioners in the early detection of cancer.

## Supporting information

S1 Text PRISMA for scoping reviews. (DOCX)

S1 Table Search strategy for Cumulative Index to Nursing and Allied Health Literature (CINHAL) (26/03/2024). (DOCX)

S2 Table Search strategy for Medline via PubMed (26/03/2024). (DOCX)

S3 Table Search strategy for PsycInfo via Ovid (26/03/2024). (DOCX)

S4 Table Search strategy for Embase (26/03/2024). (DOCX)

S2 Text Search strategy for Google Scholar. (DOCX)

S5 Table Search strategy for Open Access Theses and Dissertation (11/07/2024). (DOCX)

S6 Table Excluded articles with reason for exclusion, ordered by year of publication and first author name. (DOCX)

S7 Table Articles retrieved by ResearchRabbit we excluded with reasons for exclusion, ordered by year of publication and first author name. (DOCX)

## References

[1] Danckert B, Christensen NL, Falborg AZ, Frederiksen H, Lyratzopoulos G, McPhail S, et al. Assessing how routes to diagnosis vary by the age of patients with cancer: a nationwide register-based cohort study in Denmark. BMC Cancer. 2022;22(1):906. doi: 10.1186/s12885-022-09937-y 35986279 PMC9392355

[2] Schiff GD, Hasan O, Kim S, Abrams R, Cosby K, Lambert BL, et al. Diagnostic error in medicine: analysis of 583 physician-reported errors. Arch Intern Med. 2009;169(20):1881–7. doi: 10.1001/archinternmed.2009.333 19901140

[3] Kostopoulou O, Delaney BC, Munro CW. Diagnostic difficulty and error in primary care--a systematic review. Fam Pract. 2008;25(6):400–13. doi: 10.1093/fampra/cmn071 18842618

[4] Singh H, Giardina TD, Meyer AND, Forjuoh SN, Reis MD, Thomas EJ. Types and origins of diagnostic errors in primary care settings. JAMA Intern Med. 2013;173(6):418–25. doi: 10.1001/jamainternmed.2013.2777 23440149 PMC3690001

[5] Ferlay J, Ervik M, Lam F, Laversanne M, Colombet M, Mery L, et al. Global Cancer Observatory: Cancer Today. International Agency for Research on Cancer. 2024. https://gco.iarc.who.int/today

[6] Zakkak N, Barclay ME, Swann R, McPhail S, Rubin G, Abel GA, et al. The presenting symptom signatures of incident cancer: evidence from the English 2018 National Cancer Diagnosis Audit. Br J Cancer. 2024;130(2):297–307. doi: 10.1038/s41416-023-02507-4 38057397 PMC10803766

[7] Vecht CJ, Kerkhof M, Duran-Pena A. Seizure prognosis in brain tumors: new insights and evidence-based management. Oncologist. 2014;19(7):751–9. doi: 10.1634/theoncologist.2014-0060 24899645 PMC4077452

[8] Pearson C, Poirier V, Fitzgerald K, Rubin G, Hamilton W. Cross-sectional study using primary care and cancer registration data to investigate patients with cancer presenting with non-specific symptoms. BMJ Open. 2020;10(1):e033008. doi: 10.1136/bmjopen-2019-033008 31924638 PMC6955554

[9] Wong CJ. Involuntary weight loss. Med Clin North Am. 2014;98(3):625–43. doi: 10.1016/j.mcna.2014.01.012 24758965

[10] National Academies of Sciences, Engineering, and Medicine. Improving diagnosis in health care. Balogh E, Miller BT, Ball J, Institute of Medicine (U.S.), Editors. Washington, DC: The National Academies Press; 2015.

[11] Pocklington C. Depression in older adults. Br J Med Pract. 2017;10:a1007.

[12] Renzi C, Kaushal A, Emery J, Hamilton W, Neal RD, Rachet B, et al. Comorbid chronic diseases and cancer diagnosis: disease-specific effects and underlying mechanisms. Nat Rev Clin Oncol. 2019;16(12):746–61. doi: 10.1038/s41571-019-0249-6 31350467

[13] Cullen W, Kearney Y, Bury G. Prevalence of fatigue in general practice. Ir J Med Sci. 2002;171(1):10–2. doi: 10.1007/BF03168931 11993585

[14] Bjerager M, Palshof T, Dahl R, Vedsted P, Olesen F. Delay in diagnosis of lung cancer in general practice. Br J Gen Pract. 2006;56(532):863–8. 17132354 PMC1927095

[15] Olesen F, Hansen RP, Vedsted P. Delay in diagnosis: the experience in Denmark. Br J Cancer. 2009;101 Suppl 2(Suppl 2):S5-8. doi: 10.1038/sj.bjc.6605383 19956163 PMC2790711

[16] Neal RD, Tharmanathan P, France B, Din NU, Cotton S, Fallon-Ferguson J, et al. Is increased time to diagnosis and treatment in symptomatic cancer associated with poorer outcomes? Systematic review. Br J Cancer. 2015;112 Suppl 1(Suppl 1):S92-107. doi: 10.1038/bjc.2015.48 25734382 PMC4385982

[17] Renzi C, Odelli S, Morani F, Benitez Majano S, Signorelli C. Delays in cancer diagnosis: challenges and opportunities in Europe. Acta Biomed. 2023;94(S3):e2023161. doi: 10.23750/abm.v94iS3.14513 37695178

[18] Christensen KG, Fenger-Grøn M, Flarup KR, Vedsted P. Use of general practice, diagnostic investigations and hospital services before and after cancer diagnosis - a population-based nationwide registry study of 127,000 incident adult cancer patients. BMC Health Serv Res. 2012;12:224. doi: 10.1186/1472-6963-12-224 22838741 PMC3507912

[19] Hauswaldt J, Hummers-Pradier E, Himmel W. Does an increase in visits to general practice indicate a malignancy?. BMC Fam Pract. 2016;17:94. doi: 10.1186/s12875-016-0477-0 27456975 PMC4960682

[20] Jensen H, Vedsted P, Møller H. Consultation frequency in general practice before cancer diagnosis in relation to the patient’s usual consultation pattern: A population-based study. Cancer Epidemiol. 2018;55:142–8. doi: 10.1016/j.canep.2018.06.007 29960925

[21] Johansen M-L, Holtedahl KA, Rudebeck CE. How does the thought of cancer arise in a general practice consultation? Interviews with GPs. Scand J Prim Health Care. 2012;30(3):135–40. doi: 10.3109/02813432.2012.688701 22747066 PMC3443936

[22] Jensen E, Kristensen JK, Bjerglund RT, Johnsen SP, Thomsen JL. The pathway and characteristics of patients with non-specific symptoms of cancer: a systematic review. BMC Cancer. 2022;22(1):574. doi: 10.1186/s12885-022-09535-y 35606715 PMC9125836

[23] Langue G, Moran V, Pilleron S. Factors influencing non-specific symptom attribution to cancer in general practice: a systematic scoping review protocol. 2024. doi: 10.17605/OSF.IO/X92KD

[24] Peters MDJ, Godfrey CM, Khalil H, McInerney P, Parker D, Soares CB. Guidance for conducting systematic scoping reviews. Int J Evid Based Healthc. 2015;13(3):141–6. doi: 10.1097/XEB.0000000000000050 26134548

[25] National Institute for Health and Care Excellence. Suspected cancer: recognition and referral. London: NICE; 2023. Report No.: 12. Available from: https://www.nice.org.uk/guidance/ng12 32212590

[26] Cole V, Boutet M. ResearchRabbit (product review). J Can Health Libr Assoc. 2023;44(2):43–7. doi: 10.29173/jchla29699

[27] Walter F, Webster A, Scott S, Emery J. The Andersen Model of Total Patient Delay: a systematic review of its application in cancer diagnosis. J Health Serv Res Policy. 2012;17(2):110–8. doi: 10.1258/jhsrp.2011.010113 22008712 PMC3336942

[28] The EndNote Team. EndNote. Philadelphia, PA: Clarivate; 2013.

[29] Ouzzani M, Hammady H, Fedorowicz Z, Elmagarmid A. Rayyan-a web and mobile app for systematic reviews. Syst Rev. 2016;5(1):210. doi: 10.1186/s13643-016-0384-4 27919275 PMC5139140

[30] Wagland R, Brindle L, James E, Moore M, Esqueda AI, Corner J. Facilitating early diagnosis of lung cancer amongst primary care patients: The views of GPs. Eur J Cancer Care (Engl). 2017;26(3):e12704. doi: 10.1111/ecc.12704 28497588 PMC5949863

[31] Saab MM, O’Driscoll M, FitzGerald S, Sahm LJ, Leahy-Warren P, Noonan B, et al. Referring patients with suspected lung cancer: a qualitative study with primary healthcare professionals in Ireland. Health Promot Int. 2022;37(3):daac088. doi: 10.1093/heapro/daac088 35810412 PMC9271233

[32] Barbarit A, Guyon C, Poupin E, Frèche B. Thromboembolic disease and cancer: Attitude and practice of general practitioners. A qualitative study. Bull Cancer. 2023;110(12):1311–21. doi: 10.1016/j.bulcan.2023.08.001 37690878

[33] Hajdarevic S, Högberg C, Marzo-Castillejo M, Siliņa V, Sawicka-Powierza J, Esteva M, et al. Exploring why European primary care physicians sometimes do not think of, or act on, a possible cancer diagnosis. A qualitative study. BJGP Open. 2023;7(4):BJGPO.2023.0029. doi: 10.3399/BJGPO.2023.0029 37380218 PMC11176697

[34] Scheel BI, Ingebrigtsen SG, Thorsen T, Holtedahl K. Cancer suspicion in general practice: the role of symptoms and patient characteristics, and their association with subsequent cancer. Br J Gen Pract. 2013;63(614):e627-35. doi: 10.3399/bjgp13X671614 23998843 PMC3750802

[35] Donker GA, Wiersma E, van der Hoek L, Heins M. Determinants of general practitioner’s cancer-related gut feelings-a prospective cohort study. BMJ Open. 2016;6(9):e012511. doi: 10.1136/bmjopen-2016-012511 27625064 PMC5030540

[36] Singh H, Giardina TD, Petersen LA, Smith MW, Paul LW, Dismukes K, et al. Exploring situational awareness in diagnostic errors in primary care. BMJ Qual Saf. 2012;21(1):30–8. doi: 10.1136/bmjqs-2011-000310 21890757 PMC3692739

[37] Smith CF, Drew S, Ziebland S, Nicholson BD. Understanding the role of GPs’ gut feelings in diagnosing cancer in primary care: a systematic review and meta-analysis of existing evidence. Br J Gen Pract. 2020;70(698):e612–21. doi: 10.3399/bjgp20X712301 32839162 PMC7449376

[38] Stolper E, Van Royen P, Van de Wiel M, Van Bokhoven M, Houben P, Van der Weijden T, et al. Consensus on gut feelings in general practice. BMC Fam Pract. 2009;10:66. doi: 10.1186/1471-2296-10-66 19761589 PMC2754436

[39] Wachter RM. Why diagnostic errors don’t get any respect--and what can be done about them. Health Aff (Millwood). 2010;29(9):1605–10. doi: 10.1377/hlthaff.2009.0513 20820015

[40] Croskerry P. Perspectives on diagnostic failure and patient safety. Healthc Q. 2012;15 Spec No:50–6. doi: 10.12927/hcq.2012.22841 22874447

[41] Yao M, Kaneko M, Watson J, Irving G. Gut feeling for the diagnosis of cancer in general practice: a diagnostic accuracy review. BMJ Open. 2023;13(8):e068549. doi: 10.1136/bmjopen-2022-068549 37567752 PMC10423799

[42] Pedersen A, Vedsted P. General practitioners’ anticipated risk of cancer at referral and their attitude to risk taking and to their role as gatekeeper. J Health Serv Res Policy. 2015;20(4):210–6. doi: 10.1177/1355819615601822 26377726

[43] Pilleron S. Reducing Disparities in Cancer Outcomes. In: LIH Research Portal [Internet]. [cited 9 Jan 2025]. Available: https://researchportal.lih.lu/en/projects/reducing-disparities-in-cancer-outcomes

[44] Sarfati D, Koczwara B, Jackson C. The impact of comorbidity on cancer and its treatment. CA Cancer J Clin. 2016;66(4):337–50. doi: 10.3322/caac.21342 26891458

[45] Mounce LTA, Price S, Valderas JM, Hamilton W. Comorbid conditions delay diagnosis of colorectal cancer: a cohort study using electronic primary care records. Br J Cancer. 2017;116(12):1536–43. doi: 10.1038/bjc.2017.127 28494470 PMC5518856

[46] Boakye D, Günther K, Niedermaier T, Haug U, Ahrens W, Nagrani R. Associations between comorbidities and advanced stage diagnosis of lung, breast, colorectal, and prostate cancer: A systematic review and meta-analysis. Cancer Epidemiol. 2021;75:102054. doi: 10.1016/j.canep.2021.102054 34773768

[47] Ansah JP, Chiu C-T. Projecting the chronic disease burden among the adult population in the United States using a multi-state population model. Front Public Health. 2023;10:1082183. doi: 10.3389/fpubh.2022.1082183 36711415 PMC9881650

[48] Hacker K. The burden of chronic disease. Mayo Clin Proc Innov Qual Outcomes. 2024;8(1):112–9. doi: 10.1016/j.mayocpiqo.2023.08.005 38304166 PMC10830426

[49] Lewis R. Improving diagnostic pathways for patients with vague symptoms. United Kingdom; 2017 p. 49. Available: https://www.cancerresearchuk.org/sites/default/files/full_report.pdf

[50] Damhus CS, Siersma V, Dalton SO, Brodersen J. Non-specific symptoms and signs of cancer: different organisations of a cancer patient pathway in Denmark. Scand J Prim Health Care. 2021;39(1):23–30. doi: 10.1080/02813432.2021.1880094 33629891 PMC7971193

