# Supplementary material for: Attributing non-specific symptoms to cancer in general practice: A scoping review
Source: PLoS One. 2025 Jun 23;20(6):e0322264. doi: 10.1371/journal.pone.0322264 (PMC12184906; doi:10.1371/journal.pone.0322264)
Supplement: S2 Text — (DOCX) [file pone.0322264.s006.docx]

## S2 Text. Search strategy for Google Scholar

11/07/2024

2022- 2024: allintitle: general practitioner AND cancer diagnosis (4 results)

2022 - 2024: allintitle: general practice AND cancer diagnosis (5 results)

12/07/2024

2022 - 2024: allintitle: general practitioner AND cancer AND diagnose (0 result)

2022 - 2024: allintitle: GP AND cancer AND diagnosing (0 result)
