# Supplementary material for: Attributing non-specific symptoms to cancer in general practice: A scoping review
Source: PLoS One. 2025 Jun 23;20(6):e0322264. doi: 10.1371/journal.pone.0322264 (PMC12184906; doi:10.1371/journal.pone.0322264)
Supplement: S5 Table — (DOCX) [file pone.0322264.s007.docx]

## S5 Table. Search strategy for Open Access Theses and Dissertation (11/07/2024)

| **Num** | **Search** | **Hits** |
| --- | --- | --- |
| 8 | abstract:("general practitioner") AND abstract:(cancer) AND abstract:("diagnosis") AND pub_dt:[2022-01-01T00:00:00Z TO *] | 4 |
| 7 | title:("general practitice") AND ("cancer diagnosis") | 3 |
| 6 | abstract:(GP) AND abstract:("cancer diagnosis") AND pub_dt:[2022-01-01T00:00:00Z TO *] | 2 |
| 5 | abstract:(diagnostic AND reasoning) AND abstract:(cancer) AND abstract:("general practice") | 1 |
| 4 | abstract:("think of cancer") AND abstract:(general OR practice) AND pub_dt:[2022-01-01T00:00:00Z TO *] | 0 |
| 3 | abstract:("clinical reasoning") AND abstract:(general OR practice) AND abstract:("cancer") | 4 |
| 2 | abstract:("think of cancer") AND abstract:(general OR practice) AND pub_dt:[2022-01-01T00:00:00Z TO *] | 0 |
| 1 | abstract:(diagnostic AND reasoning) AND abstract:(cancer) AND abstract:("general practice") | 1 |
