# Supplementary material for: Attributing non-specific symptoms to cancer in general practice: A scoping review
Source: PLoS One. 2025 Jun 23;20(6):e0322264. doi: 10.1371/journal.pone.0322264 (PMC12184906; doi:10.1371/journal.pone.0322264)
Supplement: S7 Table — (DOCX) [file pone.0322264.s009.docx]

## S7 Table. Articles retrieved by ResearchRabbit we excluded with reasons for exclusion, ordered by year of publication and first author name

| **First author, date** | **Title** | **Reason for exclusion** |
| --- | --- | --- |
| Holtedahl, 1990 | Early clues to cancer | Wrong publication type |
| Holtedahl, 1991 | Diagnosis of cancer in general practice. A study of delay problems and warning signals of cancer, with implications for public cancer information and for cancer diagnostic strategies in general practice | Wrong publication type |
| Goodman, 1993 | Delay in the diagnosis and prognosis of carcinoma of the right colon | No info on diagnostic reasoning |
| Bordage, 1999 | Why did I miss the diagnosis? Some cognitive explanations and educational implications | Wrong publication type |
| Young, 2000 | Implications of delayed diagnosis in colorectal cancer | No focus on non-specific symptoms |
| Khattak, 2006 | Colorectal cancer – a prospective evaluation of symptom duration and GP referral patterns in an inner city teaching hospital | No info on diagnostic reasoning |
| Singh, 2007 | Errors in cancer diagnosis: current understanding and future directions | No focus on non-specific symptoms |
| Downs, 2008 | How people think about cancer: A mental models approach | Wrong publication type |
| Kostopoulou, 2008 | Diagnostic difficulty and error in primary care--a systematic review | No focus on non-specific symptoms |
| Heneghan, 2009 | Diagnostic strategies used in primary care | Wrong publication type |
| Schiff, 2009 | Diagnostic error in medicine: analysis of 583 physician-reported errors | No focus on non-specific symptoms |
| An, 2009 | Burden of difficult encounters in primary care: Data from the minimizing error, maximizing outcomes study | No info on diagnostic reasoning |
| Fisher, 2010 | Determinants of medical system delay in the diagnosis of colorectal cancer within the veteran affairs health system | No info on diagnostic reasoning |
| Singh, 2010 | Characteristics and predictors of missed opportunities in lung cancer diagnosis: An electronic health record–based study | No info on diagnostic reasoning |
| Buntinx, 2011 | Dealing with low-incidence serious diseases in general practice | Wrong publication type |
| Ellis, 2011 | Delays in the diagnosis of lung cancer | No info on diagnostic reasoning |

**Table S7** (continued)

| **First author, date** | **Title** | **Reason for exclusion** |
| --- | --- | --- |
| Poon, 2012 | Cognitive errors and logistical breakdowns contributing to missed and delayed diagnoses of breast and colorectal cancers: A process analysis of closed malpractice claims | No info on diagnostic reasoning |
| Abgrall-Barbry, 2012 | Depressive mood and subsequent cancer diagnosis in patients undergoing a colonoscopy | No info on diagnostic reasoning |
| Ely, 2012 | Diagnostic errors in primary care: lessons learned | No focus on non-specific symptoms |
| Ayala, 2012 | Missed opportunities in early diagnosis of symptomatic colorectal cancer | No focus on non-specific symptoms |
| Sarkar, 2012 | Challenges of making a diagnosis in the outpatient setting: a multi-site survey of primary care physicians | No focus on non-specific symptoms |
| Tsang, 2013 | Cancer diagnosed by emergency admission in England: an observational study using the general practice research database | No info on diagnostic reasoning |
| Singh, 2013 | Types and origins of diagnostic errors in primary care settings | No info on diagnostic reasoning |
| Schichtel, 2013 | Educational interventions for primary healthcare professionals to promote the early diagnosis of cancer: a systematic review | No info on diagnostic reasoning |
| Hamilton, 2013 | Easily missed? Colorectal cancer | Wrong publication type |
| Woolley, 2013 | Clinical intuition in family medicine: More than first impressions | No focus on non-specific symptoms |
| Holtedahl, 2014 | Predictive values of GPs' suspicion of serious disease are high enough to warrant subsequent investigation | Wrong publication type |
| Andersen, 2015 | The association between attachment and delay in the diagnosis of cancer in primary care | Wrong publication type |
| Lyratzopoulos | Understanding missed opportunities for more timely diagnosis of cancer in symptomatic patients after presentation | Wrong study design |
| Biswas, 2015 | Symptom lead times in lung and colorectal cancers: what are the benefits of symptom-based approaches to early diagnosis? | No info on diagnostic reasoning |
| Huo, 2015 | Delay in diagnosis and treatment of symptomatic breast cancer in China | No focus on non-specific symptoms |
| Nicholson, 2016 | Can safety-netting improve cancer detection in patients with vague symptoms? | Wrong publication type |

**Table S7** (continued)

| **First author, date** | **Title** | **Reason for exclusion** |
| --- | --- | --- |
| Robinson, 2016 | What are the factors influencing GPs in the recognition and referral of suspected lung cancer | Wrong publication type |
| Hamilton, 2016 | Improving early diagnosis of symptomatic cancer | Wrong study design |
| Mounce, 2017 | Comorbid conditions delay diagnosis of colorectal cancer: a cohort study using electronic primary care records | No info on diagnostic reasoning |
| Waller, 2018 | Diagnosing cancer in patients with 'non-alarm' symptoms: Learning from diagnostic care innovations in Denmark | Wrong publication type |
| Nicholson, 2019 | When should unexpected weight loss warrant further investigation to exclude cancer | Wrong study design |
| Renzi, 2019 | Comorbid chronic diseases and cancer diagnosis: disease-specific effects and underlying mechanisms | Wrong study design |
| Heyhoe, 2019 | Developing a safety-netting intervention for the earlier diagnosis of cancer in primary care: the Shared Safety Net Action Plan (SSNAP) | Wrong publication type |
| Summerton, 2019 | Artificial intelligence and diagnosis in general practice | Wrong publication type |
| Cassim, 2019 | Patient and carer perceived barriers to early presentation and diagnosis of lung cancer: a systematic review | No info on diagnostic reasoning |
| Surgey, 2020 | ThinkCancer! The multi-method development of a complex behaviour change intervention to improve the early diagnosis of cancer in primary care | Wrong publication type |
| Nicholson, 2020 | The association between unexpected weight loss and cancer diagnosis in primary care: a matched cohort analysis of 65,000 presentations | No info on diagnostic reasoning |
| Price, 2023 | Examining methodology to identify patterns of consulting in primary care for different groups of patients before a diagnosis of cancer: An exemplar applied to oesophagogastric cancer | No focus on non-specific symptoms |
